# Emotion Modulation of Visual Attention: Categorical and Temporal Characteristics

**DOI:** 10.1371/journal.pone.0013860

**Published:** 2010-11-05

**Authors:** Bethany G. Ciesielski, Thomas Armstrong, David H. Zald, Bunmi O. Olatunji

**Affiliations:** Department of Psychology, Vanderbilt University, Nashville, Tennessee, United States of America; University of Granada, Spain

## Abstract

**Background:**

Experimental research has shown that emotional stimuli can either enhance or impair attentional performance. However, the relative effects of specific emotional stimuli and the specific time course of these differential effects are unclear.

**Methodology/Principal Findings:**

In the present study, participants (*n* = 50) searched for a single target within a rapid serial visual presentation of images. Irrelevant fear, disgust, erotic or neutral images preceded the target by two, four, six, or eight items. At lag 2, erotic images induced the greatest deficits in subsequent target processing compared to other images, consistent with a large emotional attentional blink. Fear and disgust images also produced a larger attentional blinks at lag 2 than neutral images. Erotic, fear, and disgust images continued to induce greater deficits than neutral images at lag 4 and 6. However, target processing deficits induced by erotic, fear, and disgust images at intermediate lags (lag 4 and 6) did not consistently differ from each other. In contrast to performance at lag 2, 4, and 6, enhancement in target processing for emotional stimuli was observed in comparison to neutral stimuli at lag 8.

**Conclusions/Significance:**

These findings suggest that task-irrelevant emotion information, particularly erotica, impairs intentional allocation of attention at early temporal stages, but at later temporal stages, emotional stimuli can have an enhancing effect on directed attention. These data suggest that the effects of emotional stimuli on attention can be both positive and negative depending upon temporal factors.

## Introduction

Attention's inextricable link to emotion is implied, but not necessarily stated in cognitive theories of emotion [1]. However, a full understanding of this linkage requires increased specification of the properties of this linkage as attention is multidimensional, and can involve both intentional (top-down) directed or selective attention commensurate with goal pursuits, as well as bottom-up involuntary effects in which attention is captured by salient stimuli.

Research on emotion influences on attention has focused mainly on the prioritization of emotional stimuli over neutral stimuli using paradigms that utilize a single or restricted temporal window to examine effects. However, recent studies examining emotional influences over a longer timescale suggest that the adaptive effects of emotion on attention are more dynamic and complex than typically appreciated. For example, Bocanegra and Zeelenberg [2] manipulated the temporal distance between emotional cues (negative words) and a subsequent neutral target by varying cue-target inter-stimulus intervals (ISIs). Emotional cues impaired target identification at short ISIs (50 and 500 ms) but improved target identification at longer ISIs (1000 ms). A subsequent study using emotional auditory stimuli further supports that emotional stimuli may have a twofold effect on attention (e.g.; [3]). Whereas emotional stimuli may initially capture and hold attention, thereby impairing processing of contiguous or temporally proximal neutral stimuli, processing of neutral stimuli may be enhanced once attention is released from emotional stimuli. For example, in studies presenting a fearful face followed by a pause sufficient to allow disengagement of attention, improvements in perception (i.e. contrast sensitivity; [4]) and search efficiency [5] have been observed. These findings follow an evolutionary logic, as ‘emotion induced blindness’ may be adaptive in forcing us to register important stimuli, but would quickly become a liability if attention could not be reallocated towards other relevant information necessary for the execution of an appropriate response.

Although time course appears to dictate the extent to which emotion improves or impairs directed attention, it is not clear if the twofold effect is observed for emotion in general or if this effect varies as a function of the specific type of emotional information. For example, the twofold effects of emotion on attention have been almost exclusively reported in paradigms using either arousing, and mostly negatively valenced words or fearful facial expressions. Although other types of emotional stimuli (such as emotionally valenced pictures) have clearly been observed to impact performance on tasks requiring directed attention, it is not clear if these stimuli also produce this two-stage capture followed by enhancement effect. Also, it is unclear how different aversive contents may vary in their effects on attention across time. It has been suggested that different negative emotional contents have distinct effects on attention (e.g., [6]), and the few extant studies testing this hypothesis suggest that differential patterns exist. For example, a recent study found that while difficulty disengaging attention is observed during exposure to fear and disgust stimuli, this effect is greater for disgust stimuli compared to fear stimuli [7]. Vermeulen, Godefroid, and Mermillod [8] also found that processing fear exerts greater inhibitory responses on distractors relative to processing disgust. This may have functional significance in that observation of fear in a peer warrants quick identification, but also a rapid shift away from them to identify the source of the fear. In contrast, disgust information, relaying the likelihood of contamination, may not necessitate rapid shifting of attention, but may rather facilitate further processing or extension of the hold component of deployed attentional resources in order to fully characterize the risk. Such a model appears consistent with recent research showing that the perception of fear is gated by selective attention at early latencies during exposure, whereas the perception of disgust appears to be modulated by attention allocation at later latencies [9].

Similarly, it is necessary to determine if these effects are valence or arousal specific. In a number of paradigms, erotic stimuli produce as strong or stronger effects than aversive stimuli on task performance [10], [11]. Such findings strongly argue that many emotional effects are driven by arousal rather than valence. However, to date evidence regarding whether such positive arousing stimuli have a two-stage impact on attention is lacking.

The present study employs a rapid serial visual presentation (RSVP) paradigm to assess emotion's modulation of attention at different time intervals. Specifically, the study examines differences in attentional capture and subsequent attentional enhancement between fear, disgust, erotic and neutral images. In order to examine the two-fold effect which suggests that emotion impairs attention at shorter time intervals but improves it at longer time intervals [2], the intervals between distracter and target images were varied at 200 ms, 400 ms, 600 ms, and 800 ms lags. Examination of performance at these 4 lags represents a novel extension of prior research examining the time course of the emotional attentional blink, which has traditionally been examined at just 2 lags. It was predicted that emotional images would produce large deficits in target identification compared to neutral images. It was also predicted that deficits in target identification would be more pronounced when the irrelevant emotional images preceded the target at shorter lags relative to longer lags. With regards to time course, it was predicted that attentional enhancement effects of emotion would be observed at longer lags relative to the shorter lags of the RSVP.

## Methods

### Participants

The study was conducted with approval of the ethic committee, the Institutional Review Board of Vanderbilt University, Nashville, TN, USA. Fifty undergraduate students, each of whom gave informed written consent prior to beginning the study (76% female; 72% Caucasian) with a mean age of 19.54 (SD = 1.13) participated in exchange for research credit.

### Stimuli

The visual stimuli were images standardized to 320 by 240 pixels consisting of four categories of emotional distracters: 42 disgusting images, 42 erotic images, 42 fear evoking images, 42 neutral images, 252 upright landscapes/architectural that were used as ‘background stimuli, and 80 target images consisting of landscape/architectural photos, 40 rotated 90° degrees to the left and 40 rotated 90° to the right. Fear, disgust, and neutral pictures were partially drawn from the International Affective Picture System (IAPS; [12]) and were supplemented with similar pictures found from publicly available sources. Our partitioning of the images to the specific emotional categories was guided by prior research that has addressed this issue with the IAPS. In cases where images were supplemented with pictures found from publicly available sources, effort was made to employ only images that were representative of the specific emotional category as revealed by prior research. Fear pictures included animals bearing teeth in a threatening manner, humans brandishing weapons, and explosions. Disgust pictures were of contaminated or diseased items including roaches, feces, diseased flesh, and maggot ridden food products. Neutral pictures were scenic in style and included both animals and humans. The erotic images were of nude male-female couples engaging in sexual scenarios and were drawn from the image set used by Most and colleagues [11].

### Procedure

**Figure 1:**
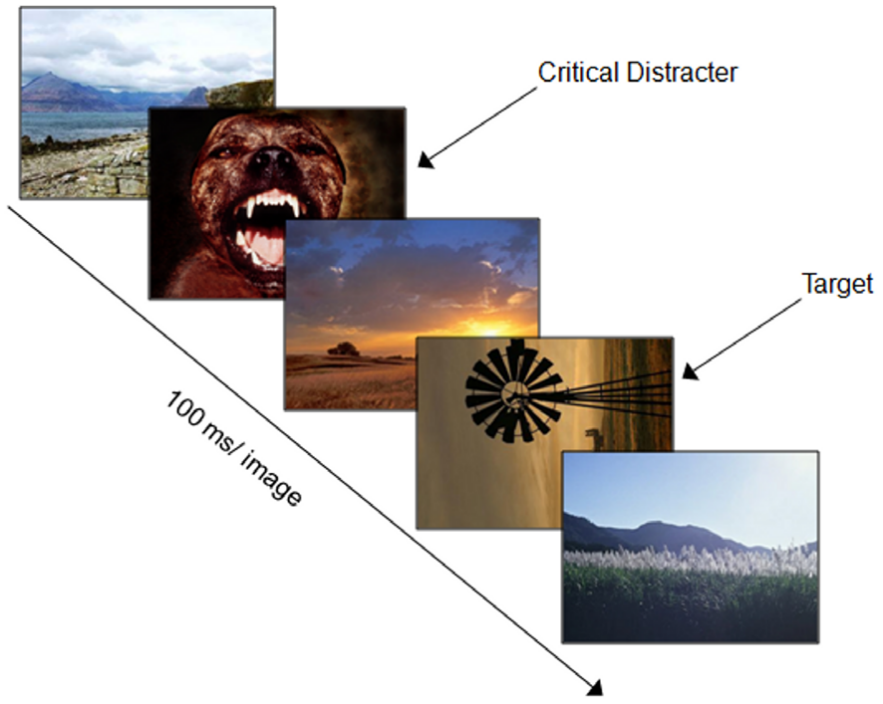
The trial procedure for the emotional attentional-blink paradigm. Note that the distractors were varied in four categories, disgust, erotic, fear, and neutral at 200, 400, 600, and 800 ms time lags.

On each trial of the RSVP task, 17 images on a white background were presented for 100 ms each and one of the images was a distracter and one of the images, the target, was rotated 90° to the left or right (see Figure 1) using E-prime Software (version 2.0, Psychology Software Tools, Inc.). The refresh rate for the monitors on which the experiment was conducted was 60 Hz, refreshed every 16.6 ms. Stimulus presentation was randomized and each trial consisted of a disgust, fear, erotic, or neutral distracter image that appeared equally in the stream at positions 4, 6, or 8; further, the distracters appeared at varying time intervals, 200 ms (lag 2), 400 ms (lag 4), 600 ms (lag 6), or 800 ms (lag 8) before the rotated image. Participants completed 6 blocks with 28 trials per block. Of the total 168 trials, each distracter type was presented 42 times with 2 trials per distracter type containing no target. The 2 no-target trials per emotion category was included as a check to ensure participants were not simply guessing at chance levels ‘yes’ and ‘no’ for seeing a target, thus if they are answering that they did see targets when none were present one might assume they are just guessing, but accuracy for disgust, erotic, fear and neutral are well above chance for target absent trials (80.28, 96.11, 91.11, & 91.39 percent correct respectively). The 4 lags were equally distributed for 40 trials with targets present per distracter type. Participants were instructed to indicate if they saw a rotated (yes, no; *detection*) image and then asked if they saw a rotated image to report which direction it was turned (right, left; *accuracy*).

## Results

### Emotion Content and Target Accuracy

A 4 (Emotion; disgust, fear, erotic, neutral) X 4 (Lag; 2, 4, 6, 8) ANOVA on accuracy revealed a significant main effect of Emotion category [*F* (3, 147) = 78.16, *p*<.001, partial η^2^ = .615]. We were initially interested in examining differences in frequency of false alarms as a function of emotional distractor. However, no such differences were observed and the overall findings for detection and accuracy were essentially identical. Analyses for accuracy, rather than detection, are presented as they reflect more precise performance on the RSVP. Bonferroni corrected pairwise comparisons revealed that the main effect of emotion was significant for erotic, disgust, and fear relative to neutral distracters (*p*s<.001). Furthermore, participants were significantly less accurate for trials of erotic distracters in comparison to both fear and disgust distracter trials (*p*s*<*.001), while fear and disgust did not differ from each other (*p* = 1.00).

### Time Course and Target Detection

The 4 (Emotion; disgust, fear, erotic, neutral) X 4 (Lag; 2, 4, 6, 8) ANOVA on percent accuracy also revealed a significant main effect of Lag [*F* (3, 147) = 276.80, *p*<.001, partial η^2^ = .850]. Bonferroni corrected pairwise comparisons showed participants to be significantly less accurate in identifying the direction of a target at Lag 2 in comparison to all other lags (*p*s<.001). Further comparisons revealed that Lag 4 was significantly worse in comparison to percent accurate for lags 6 and 8 (*p*s<.001); while Lag 6 did not differ significantly from Lag 8 for percent accuracy (*p* = 1.00).

### Emotion Content, Time Course, and Target Detection

**Figure 2:**
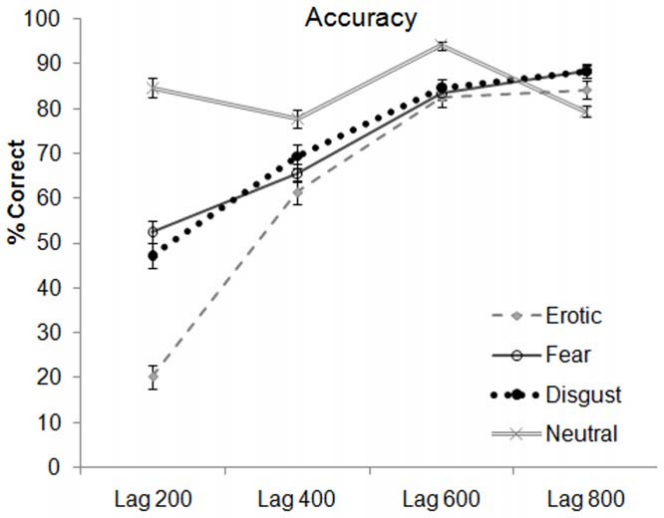
Accuracy scores for each emotion by time lag. Error bars represent standard error of the mean.

The 4 (Emotion; disgust, fear, erotic, neutral) X 4 (Lag; 2, 4, 6, 8) ANOVA on accuracy also revealed a significant Emotion X Lag interaction [*F* (9, 441) = 55.97, *p*<.001, partial η^2^ = .533]. To examine this interaction, an ANOVA on emotion at each lag was conducted. At each lag a main effect of emotion was observed (Lag 2: [*F* (3,147) = 175.285, *p*<.001, partial η2 = .782], Lag 4: [*F* (3,147) = 11.085, *p*<.001, partial η2 = .184], Lag 6: [*F* (3,147) = 12.392, *p*<.001, partial η2 = .202], Lag 8: [*F* (3,147) = 9.780, *p*<.001, partial η2 = .166]). Figure 2 shows that relative to neutral distracter trials of the same lag, participants were significantly *less* accurate in identifying the direction of the target at 200 ms, 400 ms, and 600 ms lags for all emotional distracters (*p*s<.001, *p*s<.01, *p*s<.001, respectively). Whereas, at lags of 800 ms relative to neutral trials, participants were significantly *more* accurate in identifying the target's direction for erotic, fear, and disgust distracter trials (*p*<.05; *p*<.001; *p*<.001 respectively). See Table 1 for the comparisons between each emotional distractor at each lag.

**Table 1:** Raw means and standard deviations of percent correct (accuracy) for the target for each emotional distracter category by lag interval.

	Percent Accuracy			
	Disgust	Erotic	Fear	Neutral
	M (SD)	M (SD)	M (SD)	M (SD)
Lag 2	47.33 (19.8)^c^	20.22 (19.14)^d^	52.44 (17.25)^b^	84.67 (15.37)^a^
Lag 4	69.33 (18.04)^b^	61.33 (18.41)^c^	65.56 (15.60)^bc^	77.78 (14.89)^a^
Lag 6	84.67 (13.82)^b^	82.45 (14.39)^b^	83.34 (10.82)^b^	94.00 (7.52)^a^
Lag 8	88.22 (9.37)^a^	84.22 (13.48)^b^	88.45 (10.52)^ab^	79.34 (8.98)^c^

*Note*: Means for each emotion category in the same row (same lag) with different superscripts are significantly different (all *p'*s<.05; a>b>c>d).

### Time Course of Emotional Blink Magnitude

**Figure 3:**
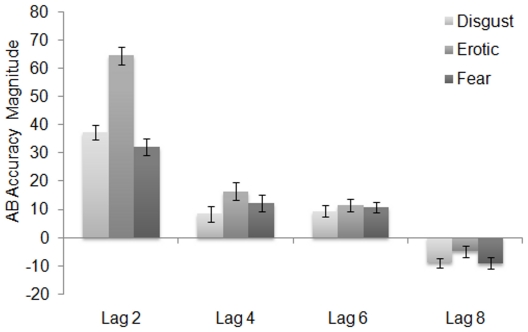
Attentional blink magnitude scores for each emotion category by lag, error bars represent standard error of the mean.

To further quantify the attentional enhancement effects of emotional, relative to neutral images, that was observed at 800 ms, an emotional blink magnitude (EBM) score was determined by subtracting the percent correct for the emotion trials at a particular lag from the neutral trials at the same lag. A positive score is reflective of a detrimental effect of emotional information on attention and a negative score is reflective of a beneficial effect on attention. A 3 (Emotion: disgust, fear, erotic) X 4 (Lag: 2, 4, 6, 8) ANOVA on the EMS revealed a significant main effect of Emotion category [*F* (2, 98) = 32.80, *p*<.001, partial η^2^ = .401], Lag [*F* (3, 147) = 129.20, *p*<.001, partial η^2^ = .725], and a Emotion X Lag interaction [*F* (6, 294) = 20.89, *p*<.001, partial η^2^ = .299]. For each emotion the comparison of the EBM between lags (i.e. Disgust Lag 2 vs. Disgust Lag 4) yielded significant differences (*p*s<.001) for all lag comparisons with the exception of lag 4 to lag 6 comparisons, which did not differ for disgust, erotic, or fear (*p* = .81; *p* = .23; *p* = .69; respectively). Table 2 presents the *t*-values for the EMB comparisons at each lag. Figure 3 shows that while the EBM at Lags, 2, 4, and 6 reflects *impairment* in the subsequent identification of a neutral target, the EBM at lag 8 reflects significant *enhancement* of the subsequent identification of a neutral target.

**Table 2:** Comparison between emotional blink magnitude scores at each lag.

	Disgust*t*-value			Erotic*t*-value			Fear*t*-value		
	Lag 2	Lag 4	Lag 6	Lag 2	Lag 4	Lag 6	Lag 2	Lag 4	Lag 6
Lag 4	9.87*	-	-	12.85*	-	-	5.83*	-	-
Lag 6	8.11*	−0.24	-	14.05*	1.21	-	6.65*	0.40	-
Lag 8	16.45*	5.88*	6.27*	18.69*	7.38*	6.14*	11.88*	6.76*	7.04*

Note:

*p<.001.

A close inspection of Table 1 and Figure 2 suggests that at the principal contrast for emotional enhancement (Lag 8), accuracy for the neutral condition appears to worsen, which suggests that the emotional enhancement effect may be an artifact of the decline in accuracy when presented with neutral distractors rather than an increase in emotional accuracy per se. Indeed, an ANOVA of accuracy for neutral distractors only revealed a main effect of Lag [*F* (3, 147) = 26.26, *p*<.001, partial η^2^ = .349]. Pairwise comparisons revealed that percent accuracy at each of the 4 lags significantly differed from each other (*p*s<.02), with the exception of Lag 2 and Lag 4 which did not differ from each other (*p* = .41). Thus, a mean neutral accuracy score across the four different Lags was computed (*M* = 83.94, *SD* = 8.49) and employed as the baseline for the contrasts with the emotional categories across the four Lags. Percent accuracy for disgust [*t* (49) = 14.72], erotic [*t* (49) = 23.73], and fear [*t* (49) = 12.87] at Lag 2 was significantly lower than the composite neutral accuracy score (*p*s<.001). Percent accuracy for disgust [*t* (49) = 6.12], erotic [*t* (49) = 8.34], and fear [*t* (49) = 8.55] at Lag 4 was also significantly lower than the composite neutral accuracy score (*p*s<.001). Although percent accuracy for the three emotional categories at Lag 6 did not significantly differ from the composite neutral accuracy score (*p*s>.37), percent accuracy for disgust [*t* (49) = −3.06] and fear [*t* (49) = −2.67] at Lag 8 was significantly higher than the composite neutral accuracy score (*p*s<.02). Percent accuracy for erotic [*t* (49) = 8.34] at Lag 8 did not significantly differ from the composite neutral accuracy score (*p* = .88).

### Are the Attentional Effects of Erotic Images Due to Valence and Arousal?

The image sets were chosen on the basis of forming coherent non-overlapping categories. In order to rule out the possibility that the unique attentional effects of erotic images were not due to differences in valence and arousal, image ratings on valence and arousal were obtained from an independent sample (*n* = 23; 65.2% female; 65.2% Caucasian) with a mean age of 20.35 (SD = 2.57). Participants rated each Disgust (valence  = −24.69, SD = 7.29; arousal  = 46.26, SD = 14.65), Erotic (valence  = 4.45, SD = 15.59; arousal  = 41.77, SD = 20.42), Fear (valence  = −15.83, SD = 7.17; arousal  = 31.98, SD  = 10.36), and Neutral (valence  = 4.87, SD = 3.66; arousal  = 6.18, SD = 5.05) image for valence (−50 =  extremely negative, +50 =  extremely positive, 0 = being no positive or negative valence/neutral) and arousal (0 =  none to 100 =  extremely/most imaginable). A significant difference for valence ratings between disgust images and all other categories was found such that disgust images were rated the most negative (*p*'s<.001). Fear images were rated as significantly more negative than erotic and neutral images (*p*'s<.001). However, the valence of erotic and neutral images did not significantly differ from each other (*p*>.90) such that both were rated on average mildly positive (above a zero score of neither positive nor negative) (although it may be noted that erotic images show a greater variance of valence ratings than any of the other stimuli with ratings). Neutral images were rated significantly less arousing than all other images (*p'*s<.001). Fear images were significantly less arousing than disgust images (*p*<.001), but not erotic images (*p*>.05). Lastly, arousal ratings for disgust and erotic images did not significantly differ from each other (*p*>.05). Given that erotic images were not uniquely characterized by differences in arousal and valance in an independent sample, it is unlikely that the attentional effects of erotic images are entirely due to unique valence and/or arousal characteristics.

## Discussion

The present findings revealed that at early time points emotional distracters produced deficits in target identification compared to neutral trials, reflecting involuntary capture of attention by emotional stimuli. This is in agreement with prior research demonstrating that emotionally laden stimuli redirect attentional resources towards emotionally salient [11], [13] and survival-relevant information [14]. Although this emotion induced ‘attentional blink’ was present for as long as 600 ms following all emotion picture types, there was a clear graded decline in the extent of the attentional blink from shorter to longer lags. This suggests processing resources captured by the emotional stimuli become increasingly available for the identification of targets over time, consistent with limited-capacity accounts of the attentional blink informed by interference models (see [15] for review). Such models suggest that the capture of attention on an item interferes with the processing of subsequent items. Accordingly, attention dwells on the initial object to inform the guidance of behavior [16], but tapers off once the potentially meaningful stimulus has been processed.

The current study highlights the importance of evaluating time course in delineating the modulation of attention by emotion. This could be seen in the attenuation of attentional blink effects at intermediate lags (400 ms and 600 ms) even for stimuli that showed initially robust emotion induced blindness. Remarkably, by 800 ms, there was no evidence of attentional blinks, but rather there was evidence of enhanced target detection following emotional stimuli relative to neutral stimuli. When accounting for variation in accuracy after neutral distractors across the four Lags, the enhancement effect was robust for fear and disgust but not erotic images. These data converge with the research of Bocanegra and Zeelenberg [2], who observed emotion-induced blindness in response to emotional words at short and intermediate ISIs, but emotion-induced “hypervision” (enhanced performance) with a longer (1000 ms) ISIs. Such enhancements at 800 or 1000 ms are in line with other recent research suggesting that negative emotion information aids successive processing of non-emotional objects under certain conditions [5].

The distinct mechanisms underlying the relative beneficial and detrimental effects of emotional information on attention over time remain unclear. It is not known whether the enhancements reflect a compensatory mechanism (engaged in response to attentional capture) or an independent process that relies on a distinct circuitry that acts on a slower time scale than attentional capture. The compensatory mechanism hypothesis would be bolstered if the extent of hypervision were proportional to the extent of the attentional capture for a stimulus group. However, the three emotional categories showed equivalent hypervision effects despite the far greater initial attentional capture by erotica (discussed below). The alternative hypothesis, that there are two distinct processes acting at different timescales, may be a better fit to the data. To the extent that distinct mechanisms account for the beneficial and detrimental carryover effects of emotion on attention, such mechanisms may take effect under a specified time course in which attentional capture predominates or even masks beneficial effects at early and intermediate time points. As such, performance at intermediate time points may not simply reflect the length of emotion induced blindness, but reflect a weighted combination of the two competing processes (especially when aggregated over individuals who likely possess differences in the strength and time course of each process).

It should be noted that the present data provides a fuller picture of the time course of emotion induced blindness than the time course of emotion induced hypervision. 800 ms is probably close to the minimum time lag for observing emotion induced hypervision, and these effects may intensify at greater delays. Indeed, the effect observed at 800 ms is relative modest relative to the robust attentional capture effects observed at briefer delays. Future research should examine the full time course of these hypervision effects.

The three emotional stimulus categories produced different levels of impact on attention depending upon the time-point examined. The greatest target identification deficits observed in the present study were for erotic stimuli at short lags. Considering their lack of harm-relevance, the strength and magnitude of the attentional blink produced by these high arousal, but overall pleasant images, is striking. This finding complements the earlier findings of Arnell and colleagues [17] and Most et al. [11], which showed that sexual content greatly diminishes RSVP task accuracy through involuntarily capture of attention. Ratings of the words used by Arnell et al. [17] suggest that arousal level, not valence, may partially account for the attentional capture by erotic stimuli. Indeed, arousal-level has also been implicated in the capture of attention by taboo words in an Emotional Stroop task [18]. However, the erotic images used in the present study were not found to be significantly more arousing than the disgust images by an independent sample. Although it is difficult to draw definitive conclusions regarding the effects of arousal and valence on the findings observed in the present study, it may be the case that arousal cannot fully explain the extent of the large attentional blink observed for erotic stimuli. Erotic images may uniquely capture attention in large part due to their “shock value.” Independent of the taboo content, erotic images may be viewed as consisting of distinct lower level physical characteristics compared to other image categories. However, a study employing similar erotic images found that scrambling erotic images, such that the basic physical properties are preserved but all meaning is lost, causes the attentional blink from erotic images to disappear (see [19]). Thus emotion content rather than low level physical characteristics of the erotic images are likely to account for erotic stimuli's advantage in capturing attentional resources at Lag 2.

Examination of the intermediate time intervals revealed that erotic stimuli's relative advantage in capturing and maintaining attentional resources diminished rapidly. Task performance observed at the 400 ms interval showed that erotic stimuli no longer caused greater target processing deficits compared to fear stimuli, and at 600 ms, erotic stimuli showed no difference from either fear or disgust stimuli. This suggests that erotic stimuli may not differ from fear or disgust with regard to the amount of time necessary for processing, or the so called hold component, which others have proposed leads to the relatively larger deficits observed following highly arousing stimuli [17]. Rather, the robust effect of erotic stimuli at lag 2 may be due to modulation of the capture component, which would show pronounced initial disruption but not necessarily prolonged ‘blindness’ for subsequent stimuli. Accordingly, erotic stimuli may initially consume the vast majority of available resources while other emotional information may initially consume only part of the available resources allowing greater processing of subsequently occurring items. Assuming that the rate of release of resources or the replenishment of resources is relatively rapid, these initial differences in capture may be largely resolved by intermediate time lags.

The present findings extend previous work by comparing the effects of different negative emotional content on attention. Prior research suggests that fear and disgust serve different functions [20], and such differences may also be revealed at the level of attention [7]. Comparisons between fear and disgust in the present investigation revealed slight differences at lag 2. However, these data do not appear to support a consistent difference in attentional capture by fear and disgust content over time. Alternatively, the overlapping harm appraisals associated with fear and disgust [21] may make it difficult to reliably detect attentional differences over time between these emotions. It is also possible that fear and disgust only differ in the capture of *spatial* attention. A cognitive task that places greater emphasis on orienting attention to identify objects might reveal distinctions between fear and disgust that were not observed in the present study.
